# Effectiveness of oral health education on 8- to 10-year-old school children in rural areas of the Magway Region, Myanmar

**DOI:** 10.1186/s12903-020-01368-0

**Published:** 2021-01-02

**Authors:** Kyu Kyu Swe, Aung Kyaw Soe, Saw Htun Aung, Htin Zaw Soe

**Affiliations:** 1 Department of Preventive and Social Medicine, Uiversity of Medicine, Magway, Myanmar; 2 Maxillo-Facial Department, Teaching Hospital, Magway, Myanmar; 3 Department of Preventive and Community Dentistry, University of Dental Medicine, Yangon, Myanmar; 4 University of Community Health, Magway, Myanmar

**Keywords:** Oral health education, Oral health knowledge and behavior

## Abstract

**Background:**

Oral diseases are common and widespread around the world. The most common oral diseases are preventable, and early onset is reversible. Myanmar faces many challenges in rendering oral health services, because approximately 70% of the total population resides in rural areas. These relate to the availability and accessibility of oral health services. Therefore, oral health education is one key element to prevent oral diseases and to promote oral health.

**Methods:**

A quasi-experimental study was carried out at Basic Education Middle Schools in rural areas of Magway Township to study the effectiveness of oral health education on the knowledge and behavior of 8- to 10-year-old school children. A total of 220 school children, 110 from intervention schools and 110 from control schools, participated in this study from 2015 to 2017. Data were collected before and after intervention in the two groups by using a self-administered questionnaire. Tooth brushing method data were collected by direct observation with a checklist. Oral health education was provided at eight weekly intervals for 1 year. At one and a half years, third-time data collection was done on the intervention group to assess retention. Chi-square test, two samples *t*-test and one-way repeated measure ANOVA were used for data analysis. The study was approved by the Institutional Review Board of the University of Public Health in Yangon, Myanmar.

**Results:**

There were significant differences between the two groups in four out of five knowledge questions (*p* < 0.05) and all behavior questions (*p* < 0.001) after intervention. A positive effect of oral health education for a period of 45 min at eight weekly intervals for 1 year was found in the intervention group. The intervention had a significant effect on the sustainability of the correct knowledge and behavior of the intervention group although the education session was stopped for 6 months (*p* < 0.001). Their mean knowledge and behavioral scores at three different points in time were (2.45 ± 1.12 and1.56 ± 0.90) at baseline, (3.79 ± 1.12 and 3.60 ± 1.21) at 1 year after education and (4.07 ± 0.98 and 3.24 ± 1.31) at 6 months after cessation of education, respectively.

**Conclusions:**

Repeated oral health education was effective in promoting and sustaining oral health knowledge and behavior.

## Introduction

Dental caries, periodontal diseases, and oral cancers are common and affect men, women, and children. Over 3.5 billion people suffer from oral diseases which continued to threaten the health, well-being, the social and economic productivity of millions of people throughout the developing world. Oral diseases are becoming global issues and should be considered as public health importance [1]. Many behavioral and social characteristics like eating habits, oral health knowledge, practices, availability, and accessibility of oral health services are some of the issues concerning oral health. Behavioral interventions or education to the individual about how to maintain the pleasant condition of a person’s mouth or how to control the mental and social problems that affect the dental health behavior are required to reduce the oral health problems and to promote oral health [2]. Personal habits like poor oral hygiene, smoking, alcohol drinking, and eating an unhealthy diet are modifiable risk factors that affect the health of gum. Periodontal disease is one of the common oral diseases and can be prevented by maintaining the individual’s good oral health behavior like tooth cleaning with a toothbrush and toothpaste, inter-dental cleaning with dental floss, and other oral hygiene measures [3]. Health education activities have a powerful effect on the behavioral characteristics of the individual like oral health knowledge, attitude, practice, eating habits, tooth decay, periodontal health, and oral hygiene [4]. It is estimated that common oral diseases such as tooth decay and gum diseases affect nearly 80% of children who are at school-going age across the globe [5]. As the lifestyle and behavioral patterns of the people are changing rapidly, these become favorable to the onset of oral diseases. Oral diseases are linked by common preventable risk factors including eating a lot of sweet food, excessive use of tobacco, high alcohol intake [5]. In Myanmar, knowledge, attitudes and practices on oral health among rural populations were low [6], and oral health status among 5-year- and 12-year- old children was not satisfactory [7]. Dental public health care services are required more than before to reduce the high level of dental caries of the 12–13-year age group in Myanmar [8]. Three-month oral health education had a positive effect on the total knowledge, attitude, and practice (KAP) scores and also plaque scores of the study group of 12-year-old Myanmar school children [9]. It is estimated that the dentist-to-population ratio in Myanmar is 1:16,000 and the dental professions are taking the responsibility to give oral health care to the whole population [10]. Besides, there are no dental therapists and dental hygienists. Hence, the Myanmar population has a low opportunity to take sufficient oral health education because of an inadequate dentist population ratio [10]. To assess the magnitude of the preventive task, it is necessary to know the oral health situation of school children. Approximately 70% of the total population in Myanmar resides in rural areas. These relate to the availability and accessibility of oral health services, and as a consequence, this may have a challenge in rendering oral health services throughout the country. Therefore, oral health education plays a pivotal role in solving oral health problems, preventing common oral diseases, and promoting the oral health of the rural population. The World Health Organization suggested that school oral health promotion activities are effective in preventing oral diseases and promoting oral health among school children [4]. In Myanmar, oral health education programs are implemented and oral health services are provided to school children yearly by a dental surgeon as part of the functions of the school health team, however, these oral health programs are not strengthened [10]. Children aged 8–10 years among school children are suitable for identifying oral health situation and for providing primary prevention because they have mixed dentition, both primary teeth and permanent teeth. Hence, the current study was planned to obtain updated information on the oral health situation of school children in Myanmar and supported the role of educational programs in promoting oral health and preventing common oral diseases at an early stage in children. Furthermore, this study was an important foundation to stimulate the development of oral health awareness among the community.

## Methods

### Study design, area, population and period

A quasi-experimental nonequivalent control group study design was carried out in two randomly selected Basic Education Middle Schools (BEMSs) in a rural area of Magway Township from 2015 to 2017 to determine the effectiveness of oral health education on oral health knowledge and behavior of 8- to 10-year-old school children. A total of 220 school children, 110 from intervention school and 110 from control school, participated in this study. 8- to 10-year-old healthy children who were attending the selected middle schools were included, and those who were unwilling to participate in the study and not present on the day of data collection were excluded.

### Sample size and sampling procedure

The sample size was calculated by n = (z_α_ + z_1-β_)^2^ (p_c_ q_c_ + p_e_ q_e_) /d^2^ + 2/d + 2 = (1.96 + 1.0364)^2^ (0.47 × 0.53 + 0.69 × 0.31)/(0.22)^2^ + 2/(0.22) + 2 with 85% power and 5% type one error rate. A drop-out rate of 10% for each group was considered. The hypothesized proportions of twice-daily tooth brushing practice after lecturing in the control group and intervention groups were 47% and 69%, respectively [11]. The sample size for each group was 110 and the total sample size was 220. Prior to conducting the study, permission was obtained from the Township Educational Officer and Township Medical Officer. Out of a total of 47 BEMSs in Magway Township, there were only four in urban areas. To obtain the required sample, in the first stage, two BEMSs from rural areas and in the second stage, 110 students from each school, were randomly selected.

### Data collection method

The research question was developed by the author based on the inputs obtained from various scientific articles and face validation was done by an independent subject expert not involved in the study following which the content validity was assessed by three experienced pediatric dental specialists in the field. A pilot survey was conducted on 30 students of the same age in one of the schools in the study area and revised it, as suggested. Attention was paid to ensure the clarity of interpretation, choice of words, and meaningfulness of the questionnaire in order to be easily apprehended by children of this age. A reliability analysis was carried out and cronbach’s alpha was 0.75. It comprised of five knowledge and five behavioral questions. The outcome was reported as correct/incorrect response to knowledge questions and proper/improper response to behavior questions. Oral health education (OHE) was given to the intervention group only at eight weekly intervals for 1 year. An oral health education session for a period of approximately 45 min was prepared on key oral health messages, such as the structure and functions of teeth, types of dentition, causes and prevention of common oral diseases, importance of brushing teeth twice daily, proper tooth brushing technique, importance of regular dental visits. Chalk and blackboard, dent form model, charts, toothbrush and toothpaste were used as oral health education aids. The proper tooth brushing technique (modified bass technique) was demonstrated on a dent form model. After completion of the whole study, an oral health education session was also conducted for the children in the control group. A visit was paid to each school before data collection to discuss the research procedure with the school headmaster, and written informed consent was obtained from the caregivers. At the beginning of the study, the baseline data were collected in both groups by using a self-administered questionnaire except for one behavioral question that is the ‘method of tooth brushing’. It was collected by direct observation with a checklist. The questionnaires, originally constructed in English and translated into Burmese (Myanmar language), were given and completed by the children under the supervision of the research team members with the help of class teachers to ensure that all questions were answered. Interpersonal communications were not allowed during answering. After a 1-year period from the collection of the baseline data, post-intervention data were collected in the two groups using the same questionnaire as at baseline. After 1 year and 6 months, retention of proper knowledge and behavior were determined in the intervention group only. Toothbrush and toothpaste were provided to all participant children in both groups before and after the intervention. The scoring system and operational definitions are shown in ‘Additional file 1: Table S1’, ‘Additional file 1: Table S2’ and ‘Additional file 1: Table S3’.

### Data management and analysis

The data were checked for completeness and consistency daily and analyzed by using SPSS version 16.0. Descriptive statistics were computed for all variables. Differences between intervention and control groups responded to the knowledge and behavior questions by correct answers before and after intervention were calculated. The net effect of the intervention program was estimated by subtracting the percentage change pre- to post-intervention in control students from that for the intervention students. One-way repeated measure ANOVA with Bonferroni correction (post hoc test) was used to determine the retention of proper knowledge and behavior on oral health at three different points in time, at baseline, at 1 year after OHE, at 6 months after cessation of OHE, in students who received OHE at eight weekly intervals for 1 year. The level of statistical significance for all tests was set at 0.05.

## Results

Table 1 shows the demographic characteristics of the school children in the two groups at baseline and 1 year after oral health education. The age distribution from 8 to 10 years before and after intervention was 19.1%, 58.2%, and 22.7% in the intervention group and 14.6%, 20.9%, and 64.5% in the control group, respectively. According to the gender, boy and girl distribution before and after intervention were 43.6% and 56.4% in the intervention group and 51.8% and 48.2% in the control group, respectively. Table 2 shows correct knowledge and proper behavior on oral health among school children between the two groups. In the intervention group, the correct proportion was higher after intervention than before regarding all knowledge questions, and in the control group, the correct response rates before and after intervention were nearly the same except for the main cause of tooth decay and gum diseases. In comparing the two groups before intervention, approximately 16% of intervention students and 12% of control students gave the true answer with regard to the main cause of tooth decay. The majority of school children in both groups gave the true answer with regard to behavior about devices used in tooth brushing before as well as after intervention. Before intervention, approximately 7% of school children in the intervention group and nearly 5% of school children in the control group used dental floss to remove food debris stuck between the teeth. Regarding the pattern of tooth brushing, nearly 5% in the intervention group and only 3% in the control group brushed their teeth according to the recommended method. Before intervention, no significant differences were found between the two groups in four out of five knowledge questions and in three out of five behavior questions (*p* > 0.05). These were knowledge about the main cause of gum diseases and behavior regarding the frequency and occasion of tooth brushing (*p* < 0.05). After intervention, significant differences were found between the two groups in four out of five knowledge questions and in all behavior questions (*p* < 0.05). The only knowledge question that showed no significant differences between the two groups was ‘foods that can cause dental caries’ (*p* > 0.05). Table 3 shows percentage changes in response to knowledge and behavior on oral health before and after intervention between the two groups, and a positive effect of oral health education for a period of 45 min at eight weekly intervals for 1 year was noted. Table 4 shows the mean knowledge and behavior scores on oral health in the intervention group only. There were 2.45 ± 1.12, 3.79 ± 1.12, and 4.07 ± 0.98 and 1.56 ± 0.90, 3.60 ± 1.21, and 3.24 ± 1.31 at baseline, 1 year after OHE and 6 months after cessation of OHE, respectively. A statistically significant effect of eight weekly intervals for 1-year OHE was found on total knowledge and behavior scores in the intervention group (*p* < 0.001). Table 5 shows highly significant differences between two different points in time (baseline vs 1 year after OHE and baseline vs 6 months after cessation of OHE) regarding total knowledge and behavior scores (*p* < 0.001) and no significant difference between 1 year after OHE and 6 months after cessation of OHE (*p* = 0.159) in knowledge and (*p* = 0.060) in behavior. It was shown that the school children in the intervention group had the ability to maintain the correct knowledge and behavior related to oral health even though the OHE session was stopped for 6 months.

**Table 1:** Demographic characteristics of school children in the intervention group (n = 110) and control group (n = 110)

Variables	Categories	Intervention group	Control group
		n (%)	n (%)
Age (year)	8	21(19.1)	16(14.6)
	9	64(58.2)	23(20.9)
	10	25(22.7)	71(64.5)
Gender	Boy	48(43.6)	57(51.8)
	Girl	62(56.4)	53(48.2)

**Table 2:** Correct knowledge and proper behavior on oral health between the two groups

Variables	Categories	Intervention	Control	*p* value
		n (%)	n (%)	
*Knowledge questions*				
The main cause of tooth decay	At baseline	17(15.5)	13(11.8)	0.432
	After 1 year	79(71.8)	20(18.2)	< 0.001
The main cause of gum diseases	At baseline	29(26.4)	64(58.2)	< 0.001
	After 1 year	60(54.5)	41(37.3)	0.010
Prevention of dental caries and periodontal diseases	At baseline	78(70.9)	85(77.3)	0.281
	After 1 year	100(90.9)	88(80.0)	0.022
Foods that can cause dental caries	At baseline	71(64.5)	72(65.5)	0.888
	After 1 year	82(74.5)	71(64.5)	0.107
Development of oral cancer	At baseline	74(67.3)	84(76.4)	0.134
	After 1 year	96(87.3)	82(74.5)	0.016
*Behavior questions*				
Frequency of tooth brushing	At baseline	39(35.5)	25(22.7)	0.038
	After 1 year	74(67.3)	7(6.4)	< 0.001
Occasion of tooth brushing	At baseline	21(19.1)	8(7.3)	0.010
	After 1 year	70(63.6)	4(3.6)	< 0.001
Devices using in tooth brushing	At baseline	99(90.0)	92(83.6)	0.163
	After 1 year	106(96.4)	84(76.4)	< 0.001
Device used to remove food debris stuck between the teeth	At baseline	8(7.3)	5(4.5)	0.391
	After 1 year	51(46.4)	4(3.6)	< 0.001
Pattern of tooth brushing (by direct observation)	At baseline	5(4.5)	3(2.7)	0.721
	After 1 year	95(86.4)	6(5.5)	< 0.001

**Table 3:** Percentage change in responses to knowledge and behavior on oral health among the school children in both groups before and after intervention

Knowledge and behavior questions on oral health	%Difference between before and after intervention		Net effect of intervention (% change)
	Intervention	Control	
*Knowledge questions*			
The main cause of tooth decay	+ 56.37	+ 6.36	+ 50.01
The main cause of gum diseases	+ 28.19	− 20.91	+ 49.1
Prevention of dental caries and periodontal diseases	+ 20	+ 2.73	+ 17.27
Foods that can cause dental caries	+ 10	− 0.9	+ 10.9
Development of oral cancer	+ 20	− 1.81	+ 21.81
*Behavior questions*			
Frequency of tooth brushing	+ 31.82	− 10.91	+ 42.73
Occasion of tooth brushing	+ 44.55	− 3.63	+ 48.18
Devices using in tooth brushing	+ 6.36	− 7.28	+ 13.64
Device used to remove food debris stuck between the teeth	+ 39.09	− 0.91	+ 40.00
Pattern of tooth brushing (by direct observation)	+ 81.81	+ 2.72	+ 79.09

**Table 4:** Oral health scores at three different points in time in the intervention group (n = 110)

Variables	Categories	Mean ± SD	*p* value
Knowledge scores	At baseline	2.45 ± 1.12	< 0.001
	At 1 year	3.79 ± 1.12	
	At one and a half years	4.07 ± 0.98	
Behavior scores	At baseline	1.56 ± 0.90	< 0.001
	At 1 year	3.60 ± 1.21	
	At one and a half years	3.24 ± 1.31	

SD, standard deviation

**Table 5:** Retention of oral health knowledge and behavior in the intervention group (n = 110)

Variables	Categories	Mean	95% CI for diff		*p* value
		diff	Lower	Upper	
Knowledge scores	Baseline vs 1 year	− 1.345	− 1.69	− 1.00	< 0.001
	1 year vs one and a half years	− 0.282	− 0.63	0.07	0.159
	Baseline vs one and a half years	− 1.627	− 1.98	− 1.28	< 0.001
Behavior scores					
	Baseline vs 1 year	− 2.04	− 2.41	− 1.67	< 0.001
	1 year vs one and a half years	0.36	− 0.01	0.74	0.060
	Baseline vs one and a half years	− 1.67	− 2.05	− 1.30	< 0.001

diff, difference; CI, confidence interval; vs, versus

## Discussion

At the beginning of the study, the minimum age of the school children in both groups was 8 years and the maximum age was 10 years. The duration of the study lasted for one and a half years. There was no attrition in either group after intervention. On the other hand, some oral health intervention studies reported that there was drop-out of the participants when assessing the effect of OHE on oral health knowledge and behavior in Wuhan City of China [12], Tehran of Iran [13], and Riyadh of Saudi Arabia [14] which are a contrast to the findings of the present study. The result of China documented that the drop-out rate is a small amount and there is no problem in assessing the outcomes [12]. The correct response rates were more or less the same between the two groups before intervention in almost all of the knowledge questions (*p* > 0.05) except one question concerning the main cause of gum diseases, in which the correct answer rate of control students was significantly greater than that of intervention students (*p* < 0.001). It may be possible that even in the absence of health education, some children might have tried to search and obtain correct answers and gain knowledge through various sources, such as social media, TV, toothpaste advertisements, etc. After a 1-year intervention, significant differences were observed between the two groups in almost all knowledge questions (*p* < 0.05) except one question concerning foods that can cause dental caries (*p* > 0.05). This may be attributed to the school co-curriculum wherein some general information about the unhealthy effect of sweetened foods and drinks on teeth is taught to the school children in the primary classes. No significant differences were found between the two groups before OHE in three out of five behavioral questions (*p* > 0.05), and with regard to frequency and occasion of tooth brushing, significantly more of the students in the intervention group brushed their teeth twice per day and cleaned their teeth in the morning before breakfast and at night before going to bed compared with their control counterparts (*p* < 0.05). This might be due to unequal accessibility and availability of dental health services among the students. However, the proportion of correct behavior was significantly higher in all behavioral items for the intervention group following OHE (*p* < 0.001). This may be because of the methods applied and the materials used in the OHE session. The results of this study are seemed to reaffirm the findings of a study conducted with samples of 1661 female primary school children in Saudi Arabia who are from 6 to 8 years old to assess the effectiveness of oral health education intervention on oral health knowledge and behavior in which there was a significant improvement in all knowledge and behavior questions after intervention (*p* < 0.001) [14]. A study conducted in China to assess the effect of school-based OHE intervention on children, mothers and school teachers reported that children in the experimental group are more than those in the control group regarding the adoption of regular oral health behavior such as tooth brushing at least twice a day, dental visits annually, use of fluoride toothpaste and less frequent consumption of cakes/biscuits which supports the present study [12]. The present study is similar to one study in Bangladesh which showed that overall significant improvement was observed in almost all the indicators of knowledge and behavior after OHE compared to before (*p* < 0.001) [15]. When the present study assessed the percentage changes in response to knowledge and behavior on oral health before and after intervention between the two groups, a positive net effect of intervention was observed. The findings of the present study were in accordance with an intervention study conducted in Ireland wherein an oral health intervention for 6 weeks was performed among primary school children aged 7–12 years and positive changes were observed in oral health knowledge and behaviors [16]. Other studies performed in Chandigarh, Northern India [17], Tanzania [18] and Greece [19] reported that school-based OHE programs significantly improved knowledge and behavior. In a study performed in Kyauktan and Tharkayta Townships of the Yangon Region in Myanmar, significant improvement of knowledge, attitude and practice scores on oral hygiene was found between the baseline and 3 months after intervention among 12-year-old school children [9]. In India, a systematic review was conducted in a total of 40 articles to assess the effectiveness of oral health education programs on knowledge, attitude, practice and oral health status. In their review, they reported that oral health education was effective in improving knowledge on oral health in all studies; however, with regard to practice outcome, thirteen studies were found to be effective and two studies were not effective [20]. Another systematic review was conducted in a total of 18 articles to evaluate the effectiveness of school dental health education on oral health status, oral health-related knowledge and practice of 6 to 12-year-old children in which OHE had a positive impact on oral health status, knowledge and practice, such as frequency and duration of brushing, use of fluoride toothpaste [21]. These disparities might be due to differences in the target age group, methods and duration of the oral health education program and background characteristics of the study subjects. The present study showed that the eight weekly oral health educations for 1 year had a statistically significant effect on total knowledge and behavior scores of the oral health among the school children in the intervention group even though stopping of the education program for 6 months after 1-year OHE, and it was found that the students in the intervention group had sustainability on positive knowledge and behavior (*p* < 0.001). Similar to the present study, a study performed in India documented that reinforcement through repeated OHE sessions in the intervention schools resulted in significant improvement of oral health knowledge and practice even after cessation of the program [22]. Another study performed in northwest England reported that schools with more frequent exposures to the program had better scores than schools with fewer exposures [23]. A study in Karachi of Pakistan showed that one-time teacher-led OHE was ineffective compared to repeated and reinforced OHE in improving oral health knowledge, behavior and oral hygiene status [24]. Hence, it can be suggested that OHE is a feasible way to reach out to all sections of the children whether rich or poor, near or far, developed or underdeveloped. The provision of OHE services improves the oral health of the students which will be passed on to their family members and neighboring community and has had an effect on the whole community of the country. The results of this study can be generalized to school children in Myanmar because schools and students are randomly selected in collecting the data for measuring the outcome variables. However, the study procedure had some limitations. Teachers and caregivers were not included in the OHE sessions, which might have affected the effectiveness of OHE since they have daily contact with the students and may be essential for the achievement of long-term benefits.

## Conclusions

In conclusion, the results indicated that repeated oral health education comprising lecturing with interactive talk, demonstration and supervised tooth brushing methods at eight weekly intervals for 1 year was found to be effective in promoting and sustaining correct knowledge and behavior among school children.

## Supplementary information

**Additional file 1.** **Table S1.** Scoring system for knowledge questions on oral health. **Table S2.** Scoring system for behavioral questions on oral health. **Table S3.** Operational definitions for the variables in the study.

## Data Availability

Data are available upon request by coauthors and reviewers.

## Abbreviations

SPSS: Statistical Package for Social Science
OHE: Oral health education
vs: Versus
