# Supplementary material for: Effectiveness of oral health education on 8- to 10-year-old school children in rural areas of the Magway Region, Myanmar
Source: BMC Oral Health. 2021 Jan 2;21:2. doi: 10.1186/s12903-020-01368-0 (PMC7777401; doi:10.1186/s12903-020-01368-0)
Supplement: Supplementary file 1 — Additional file 1. Table S1. Scoring system for knowledge questions on oral health. Table S2. Scoring system for behavioral questions on oral health. Table S3. Operational definitions for the variables in the study. [file 12903_2020_1368_MOESM1_ESM.docx]

**Additional file 1: Table S1** Scoring system for knowledge questions on oral health

| No. | Knowledge questions on oral health | Score |
| --- | --- | --- |
| (1) | The main cause of tooth decay is  1. Eating lots of meat  2. Acid from dental plaque and food debris  3. Destruction of tooth substance by insect like germs  4. Don’t know | 0  1  0  0 |
| (2) | The main cause of gum diseases is  1. Biting on hard foods  2. Excessive eating of sweet foods  3. Plaque accumulated on teeth/gums due to neglect of oral hygiene  4. Don’t know | 0  0  1  0 |
| (3) | Dental caries and periodontal diseases can be prevented by one of the following means  1. Taking vitamin tablets  2. Regular tooth brushing and good oral hygiene practices  3. Can’t be prevented, because they are hereditary  4. Don’t know | 0  1  0  0 |
| (4) | Foods that can cause dental caries  1. Fresh fruits & vegetables  2. Sweet sticky foods and soft drinks  3. Meat/fish  4. Don’t know | 0  1  0  0 |
| (5) | Excessive chewing of betel quid/smoking/drinking alcohol can destroy oral health, ultimately can develop oral cancer  1. Yes  2. No  3. Don’t know | 1  0  0 |
|  | *Minimum score = 0, Maximum score = 5, Non-response = 0* |  |

**Additional file 1: Table S2** Scoring system for behavioral questions on oral health

| No. | Behavioral questions on oral health | Score |
| --- | --- | --- |
| (1) | How many times did you brush your teeth, yesterday?  1. I miss to brush  2. Once  3. Twice  4. More than two times | 0  0  1  0 |
| (2) | At what time of the day, do you brush your teeth?  1. Morning, before breakfast  2. After breakfast and before going to bed  3. Before breakfast and before going to bed  4. During bath  5. Others, please specify ______________ | 0  1  0  0  Acceptable=1 |
| (3) | What do you use to brush your teeth?  1. Salt with fingers  2. Ash/charcoal with fingers  3. Toothbrush and tooth paste  4. Others, please specify ______________ | 0  0  1  Acceptable=1 |
| (4) | What will you do if food debris stuck between your teeth?  1. Use toothpick  2. Use dental floss  3. I will leave it as it is  4. Others, please specify ______________ | 0  1  0  Acceptable=1 |
| (5) | In which pattern do the children brush him/her teeth? (by direct observation with a checklist)  1. Horizontally for every teeth  2. Vertically from the gums toward tooth surfaces, and horizontally on grinding surfaces  3. Various directions, according to the ease of manipulation  4. Other (specify) ________________ | 0  1  0  Acceptable=1 |
|  | *Minimum score = 0, Maximum score = 5, Non-response = 0* |  |

**Additional file 1: Table S3** Operational definitions

| Variables | Operational definitions |
| --- | --- |
| Age | It is the completed age in years at him/her last birthday. |
| Sex | It refers to the biological gender of the children and is categorized as male or female. |
| Oral health | It is concerned with functional efficiency not only of teeth and structures but also of the surrounding parts of the oral cavity and the various structures related to the mastication and the maxilla-facial complex (WHO, 1965). |
| Oral health knowledge | The fact or condition of knowing oral health with familiarity gained through education or experience or association. Knowledge question consists of causes and prevention of common oral diseases, foods that can cause dental caries, betel chewing, smoking, and alcohol drinking can destroy oral health. There are 5 questions for oral health knowledge. For each question, the correct answer score is 1 mark, incorrect answer score and none answer score are 0 marks. Minimum and maximum scores are 0 and 5. |
| Oral health behavior | The school children conduct the correct practice on oral health. The behavioral question consists of frequency of tooth brushing, the occasion of tooth brushing, materials used for tooth brushing, method of tooth brushing, a device used to remove food debris stuck between the teeth. There are 5 questions for oral health behavior. For each question, the correct answer score is 1 mark, incorrect answer score and none answer score are 0 marks. Minimum and maximum scores are 0 and 5. |
| Retention | In this study, it refers to the ability of school children to sustain the correct knowledge and behavior related to oral health even though the education program is stopped. |
